# Relationships between brain-derived neurotrophic factor, clinical symptoms, and decision-making in chronic schizophrenia: data from the Iowa Gambling Task

**DOI:** 10.3389/fnbeh.2014.00417

**Published:** 2014-12-04

**Authors:** Hikaru Hori, Reiji Yoshimura, Asuka Katsuki, Kiyokazu Atake, Jun Nakamura

**Affiliations:** Department of Psychiatry, University of Occupational and Environmental HealthKitakyushu, Japan

**Keywords:** brain-derived neurotrophic factor, decision-making, schizophrenia, gambling task, cognition, depression

## Abstract

The levels of brain-derived neurotrophic factor (BDNF) are significantly decreased in patients with schizophrenia and correlate with impairments in cognitive function. However, no study has investigated the relationship between the serum BDNF levels and decision-making. We compared patients with schizophrenia to healthy controls with respect to their decision-making ability and serum BDNF levels. Eighty-six chronic schizophrenia patients and 51 healthy controls participated in this study. We controlled for gender, age, and estimated intelligence quotient (IQ), and we investigated the differences in decision-making performance on the Iowa Gambling Task (IGT) between the schizophrenia patient and control groups. We also compared the IGT scores, the serum BDNF levels, and the clinical symptoms between the groups. The IGT scores of the schizophrenia patients were lower than those of the controls. A negative correlation was detected between the mean net scores on the trials in the final two blocks and the serum BDNF levels (*p* < 0.05). Multiple regression analysis revealed that depressive symptoms and the serum BDNF levels were significantly associated with the mean net scores on the trials in the final two blocks. Based on these results, impaired sensitivity to both reward and punishment is associated with depressive symptoms and reduced serum BDNF levels in chronic schizophrenia patients and may be related to their poor performance on the IGT.

## Introduction

Brain-derived neurotrophic factor (BDNF), is a member of neurotrophins involved in growth, differentiation, maturation, and survival in immature neurons. In mature neurons, it plays an important role in synaptic plasticity, augmentation of neurotransmission and regulation of receptor sensitivity (Numakawa et al., 2010). BDNF and its high affinity receptor TrkB are widely expressed in developing and adult nervous system, and BDNF is the most abundantly expressed neurotrophic factor in the central nervous system (Balaratnasingam and Janca, 2012). Recent research has provided evidence for the contribution of BDNF to the pathophysiology of schizophrenia. Studies of the BDNF Val66Met (rs6265) showed that the Met allele is associated with lower levels of BDNF secretion and with abnormal hippocampal structure and function, providing evidence for the direct involvement of BDNF in schizophrenia (Egan et al., 2003).

Recent advances in clinical neuroscience indicate that the hippocampus and the orbitofrontal cortex (OFC) play a critical role in complex decision-making processes (Rolls, 1999; Krawczyk, 2002; Johnson et al., 2007; Yu and Fank, 2014). Patients experiencing damage to the hippocampus and the OFC exhibit striking deficits in real-life decision-making, especially social or emotional decision-making, in the context of generally well-preserved intellectual functioning. In addition, growing evidence demonstrates that schizophrenia patients exhibit emotional disturbances and social dysfunction (Mandal et al., 1999; Kohler et al., 2000; Chemerinski et al., 2002), which could be partially explained by impaired decision-making. This impairment in decision-making may occur during interpersonal interactions and social situations (Damasio, 1994). In general, decisions are made based on the assessment of reward and punishment outcomes using both cognitive and affective information (Solms and Turnbull, 2004).

The Iowa Gambling Task (IGT) was developed to assess the role of affective information in decision-making (Bechara et al., 1994). In this task, subjects are presented with four decks of cards and are asked to select any deck in any sequence, and then to take a card from it. The subjects win or lose money with each turn of a card. The participants do not appear to understand the contingencies of the game at the onset. Nevertheless, they can quite rapidly develop a “feeling,” or “hunch” in the absence of conceptual awareness.

Because cognitive dysfunction is associated with schizophrenia, we hypothesized that the serum BDNF levels are associated with the IGT scores of the chronic schizophrenia patients.

## Materials and methods

### Subjects

Eighty-six chronic schizophrenia outpatients recruited from the University of Occupational and Environmental Health participated in the present study and met the following inclusion criteria: (1) aged 20–60 years; (2) chronic illness without acute exacerbation; and (3) continuously receiving a stable dose of antipsychotics for at least 3 months. The exclusion criteria were: (1) any comorbid central nervous system disorder; (2) severe psychotic symptoms; (3) meeting the DSM-IV criteria for alcohol or other substance dependence; (4) meeting the DSM-IV criteria for mental retardation; (5) receiving antidepressants; (6) treatment with electroconvulsive therapy in the 6 months preceding the study; (7) receiving clozapine; and (8) inability to understand the study protocol. The diagnosis of schizophrenia was established based on the Structured Clinical Interview for DMS-IV (SCID) (First et al., 1996) and a comprehensive review of the patients' medical records. All patients met the criteria for schizophrenia. None were comorbid with any other psychiatric disorders. Seventy-eight of the schizophrenia patients were receiving stable dose of one antipsychotic drug (risperidone, olanzapine, quetiapine, aripiprazole, blonanserin, or perospirone). The remaining schizophrenia patients were receiving at least two antipsychotic drugs. Regarding other medications, nine patients were taking stable dosages of mood stabilizer (lithium, valproic acid, or carbamazepine), five were taking stable dosages of anticholinergic drugs and two were taking antidepressant (paroxetine or mirtazapine). Schizophrenic symptoms were rated using the Positive and Negative Syndrome Scale (PANSS) (Kay et al., 1987).

Additionally, we recruited 51 healthy volunteers (26 females and 25 males) for the healthy control group. The healthy controls consisted of individuals matched to the patients with respect to age, gender, and estimated intelligence quotient (IQ). The healthy controls had not experienced a head injury and did not suffer from any neurologic, psychotic, mood, or substance use disorder as evaluated by the SCID.

### Intelligence test

The IQ of the participants was estimated using the Japanese Adult Reading Test (Matsuoka et al., 2002; Hori et al., 2008), a Japanese version of the National Adult Reading Test (Nelson and Wilson, 1991), and those individuals exhibiting estimated IQ scores of less than 80 were excluded from this study.

### IGT

Decision-making ability was assessed using the computerized version of the IGT in Japanese (Bechara et al., 1994, 1999, 2005). Decks of cards labeled “A,” “B,” “C,” or “D” were placed in front of the subjects from left to right. Initially, 200,000 yen were given to each subject. The subjects were told that (1) they are to draw one playing card from one of the four decks on each turn, (2) this game involves betting across multiple turns, (3) they receive money every time that they draw a card but that a penalty is occasionally applied, and (4) the objective of this game is to maximize the amount of money that they have. When selecting a card, the subjects can draw a card from any of the decks and can change their selection any time as many times as they choose. The game ended at the 100th draw of a card by a subject, but the subjects were not informed about this rule beforehand. The subjects received a reward each time they drew a card; if they selected a card from the deck A or B, a reward of 10,000 yen was applied, and if they selected a card from the deck C or D, a reward of 5000 yen was applied. Simultaneously, a penalty is applied; decks A and B are referred to as “bad decks” because the immediate reward at the time of the draw is high but the penalty is also high and frequent; therefore, the player ultimately loses money as cards are drawn from these decks. Alternatively, decks C and D are referred to as the “good decks” because the immediate reward is low but the frequency and amount of the penalty is low; therefore, players who draw cards from these decks ultimately earn money. Additionally, decks A and C are categorized as “low magnitude decks” in which a low penalty is applied at a relatively high frequency, whereas decks B and D are categorized as “high magnitude decks” in which a high penalty is applied at a relatively low frequency. The task ended after 100 selections. Neither the risks of rewards or penalties for each deck nor the number of selections allowed was disclosed to the subjects. The composition of the final score and the total amount of money held by each subject at the end of the task was not disclosed to the subjects. This score represented the extent to which socially valuable resources had been increased and may also indicate the amount of risk that the subject was willing to accept given that they may have continued to lose. The frequency of shifting between advantageous (C and D) and disadvantageous (A and B) decks by the subject was computed for every 20 cards, for a total of 5 blocks.

This examination is an exercise in which the subjects are rewarded as well as occasionally penalized with each draw of a card. The subjects can learn the types of rewards and penalties that are applied and can evaluate and change their selections during the process of the game. In this examination, the subjects must use cognitive processing to predict outcomes associated with their selection of cards and generate future predictions using a complex set of results and repeated decisions.

### BDNF measurement

All blood samples were obtained between 7:00 and 10:00 a.m. in the morning fasting. Fifteen milliliters of venous blood was drawn with subjects in the supine position, after the subjects had been lying at rest overnight.

The serum BDNF levels were measured using a BDNF Emax Immunoassay Kit (Promega, Madison, WI, USA) according to the manufacturer's instructions. In short, 96-well microplates were coated with an anti-BDNF monoclonal antibody and incubated at 4°C for 18 h. The plates were incubated in a blocking buffer for 1 h at room temperature. The samples were diluted 100 times with assay buffer, and BDNF standards were maintained at room temperature under horizontal shaking for 2 h, followed by washing with the appropriate washing buffer. The plates were incubated with anti-human BDNF polyclonal antibody at room temperature for 2 h and washed with the washing buffer. Then, the plates were incubated in an anti-IgY antibody conjugated to horseradish peroxidase for 1 h at room temperature, followed by incubation in peroxidase substrate and a tetramethylbenzidine solution to induce a colorized reaction. This reaction was stopped using 1 mol/L hydrochloric acid. The absorbance at 450 nm was measured using an Emax automated microplate reader. The measurements were performed in duplicate. The standard curve was linear from 5 to 5000 pg/mL, and the detection limit was 10 pg/mL. The intra- and inter-assay coefficients of variation were 5 and 7%, respectively. The recovery rate of exogenously added BDNF to the measured plasma samples was more than 95%.

Written informed consent was obtained from all of the subjects who participated in this study. The study protocols were approved by the Ethics Committee of the University of Occupational and Environmental Health and included standard procedures for clinical research involving vulnerable participants in Japan. This study was performed according to the ethical standards of the Declaration of Helsinki. If a participant exhibited a compromised ability to consent, we excluded this individual from this study. All participants who declined to participate or otherwise did not participate were eligible for treatment and were not disadvantaged in any other manner because of their lack of participation in this study.

### Statistical analysis

The differences in the clinical variables between the groups were assessed using the *t*-test and the χ^2^ test for parametric data or using the Mann–Whitney *U*-test and the Fisher exact test for non-parametric variables. Data analysis of the IGT outcome variables was conducted using *t*-tests and repeated-measures analysis of variance. A multiple linear regression was employed to analyze the effect of the serum BDNF levels on the IGT scores while adjusting for confounding factors [depression score on the PANSS, age, estimated IQ, Chlorpromazine-equivalent (CPZ-eq), and PANSS-T score]. The correlations between the PANSS scores, the IGT scores, and the serum BDNF levels were evaluated using Pearson's correlation analysis. *P*-values of <0.05 were considered to be significant. The data were analyzed using stata13.1 software for Windows.

## Results

The demographic characteristics of the subjects are summarized in Table 1. No significant differences in age, gender, estimated IQ, or the serum BDNF levels were detected between the two groups.

**Table 1:** **Demographic and clinical information of the schizophrenia patients and the healthy control group**.

	**Healthy control**	**Schizophrenia group**	***p*-value**
Age (year)	36.7 ± 9.9	35.1 ± 12.1	0.43
Gender (M/F)	25/26	43/43	0.91
Education (years)	13.4 ± 2.2	12.7 ± 2.7	0.15
Estimated IQ	101.8 ± 7.6	99.4 ± 8.2	0.09
Duration of the illness (years)		11.4 ± 13.2	
**SCHIZOPHRENIA DIAGNOSIS**			
Paranoid type		47	
Disorganized type		27	
Catatonic type		5	
Indifferenciated type		7	
PANSS-P		17.2 ± 4.7	
PANSS-N		20.4 ± 4.6	
PANSS-G		33.2 ± 7.0	
PANSS-T		70.7 ± 11.7	
CPZeq of total antipsychotic drugs(mg/day)		470.1 ± 293.0	
Serum BDNF(ng/ml)	14.1 ± 7.3	11.8 ± 7.0	0.06

*PANSS-P, PANSS positive score; PANSS-N, PANSS negative score; PANSS-G, PANSS general psychopathological score; PANSS-T, PANSS total score; CPZ-eq, chlorpromazine-equivalent*.

### Gambling Task performance

Descriptive data for the performance on the IGT are presented in Table 2. The schizophrenia patients displayed significantly smaller difference scores on the advantageous minus disadvantageous deck selection index and earned significantly less money than the controls.

**Table 2:** **Iowa Gambling Task performance of the schizophrenia patients and the healthy control group**.

	**Healthy control**	**Schizophrenia**	***t***	***df***	***p***
Mean amount of money earned (yen)	219902.0 ± 72770.1	162348.8 ± 72369.9	4.48	135	<0.001
No. of cards chosen from deck A	17.7 ± 6.9	18.6 ± 6.0	−0.75	135	0.44
No. of cards chosen from deck B	26.0 ± 12.2	34.5 ± 11.1	−4.1	135	<0.001
No. of cards chosen from deck C	30.7 ± 12.6	19.6 ± 6.1	5.89	135	<0.001
No. of cards chosen from deck D	25.2 ± 8.9	27.2 ± 9.0	−1.21	135	0.23
Choice advantageous minus disadvantageous decks	12.2 ± 32.6	−6.3 ± 22.3	−3.6	135	<0.001

**Figure 1:**
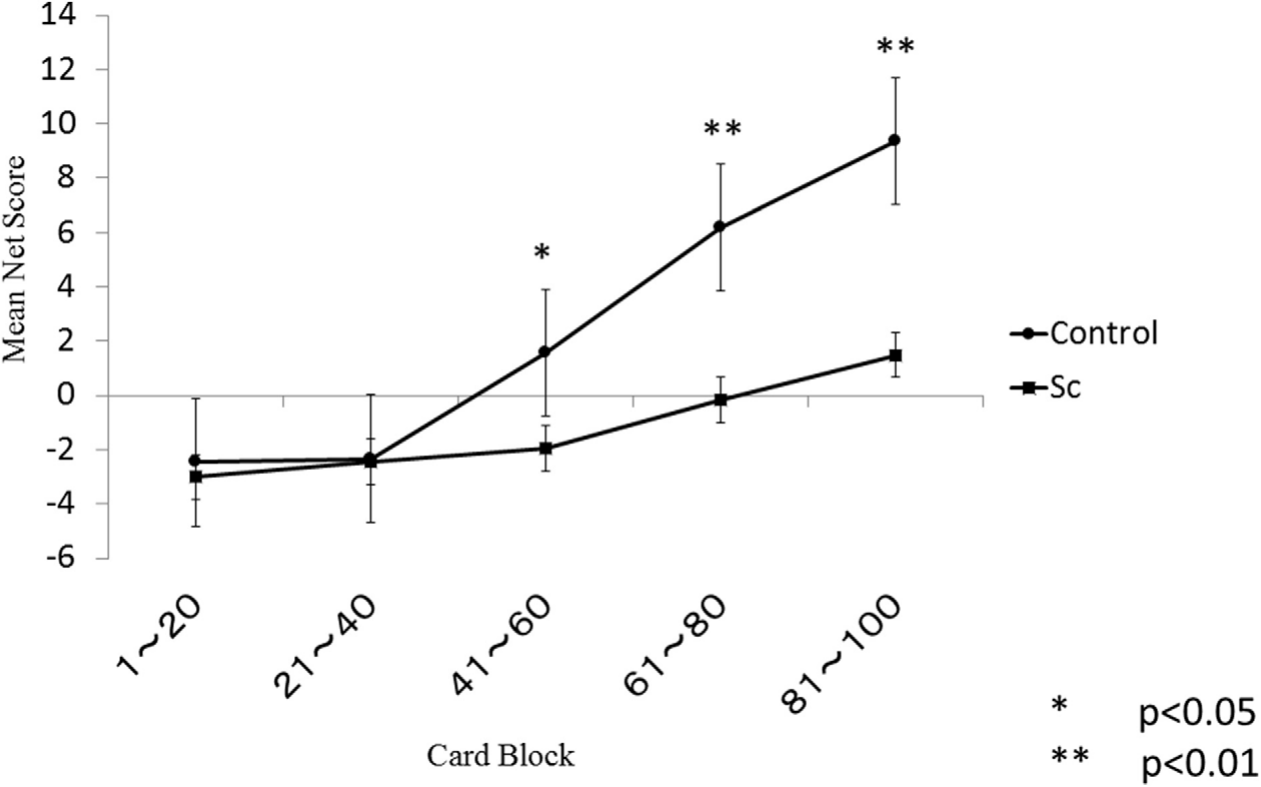
**Iowa Gambling Task in schizophrenia and healthy subjects**.

The learning curves of each group are shown in Figure 1. There were significant main effects of the group (*p* < 0.001) and the block (*p* < 0.001) and a significant group × block interaction (*p* < 0.001). A follow-up independent *t*-test indicated that the controls performed better than the schizophrenia patients during the final three blocks but not during the first two blocks. Even after correction for multiple comparisons, this between-group difference remained statistically significant for the final three blocks.

**Figure 2:**
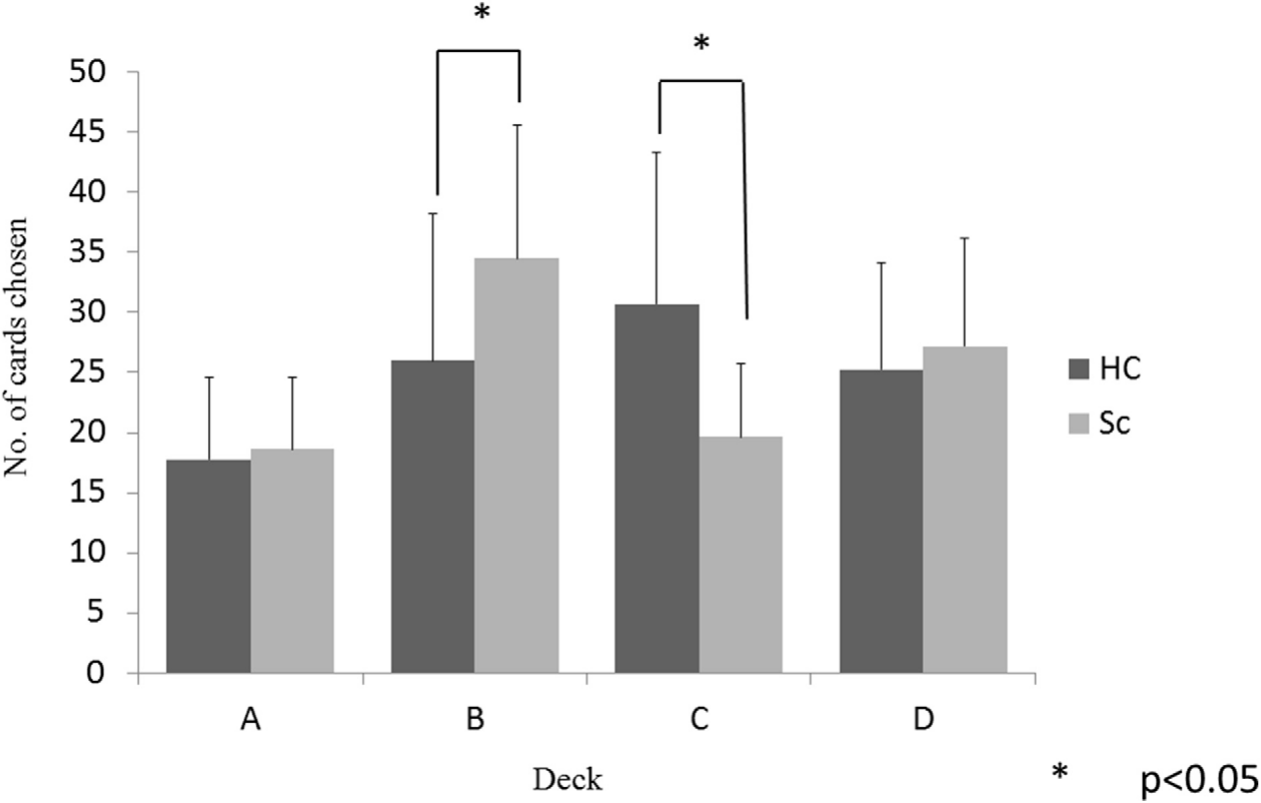
**Number of card choice selected from each deck during the 100-card task**.

The between-group differences in selections from each deck were examined using a 2 (group) × 4 (deck) repeated-measures ANOVA. There was a significant main effect of the deck (*p* < 0.001) and a significant group × deck interaction (*p* < 0.001). Follow-up *t*-tests evaluated the between-group differences in the selection from each deck. As shown in Figure 2, the schizophrenia patients selected deck B more frequently and deck C less frequently than the controls, whereas the two groups did not differ in the selection of decks A and D (*p* < 0.05, even after the Bonferroni correction). Within-group comparisons were used to clarify the pattern of performance; the controls selected the advantageous decks more frequently [(C + D) − (A + B)], (*t* = 3.95, *p* < 0.001) than the patients with schizophrenia.

### Gambling Task performance, clinical symptoms, and serum BDNF levels

No correlation was found between the total scores and mean net scores on the IGT and the PANSS-P, PANSS-N, or PANSS-T (Table 3). A significant negative correlation was detected between the mean net scores on trials 61–100 and the PANSS-G scores. A significant negative correlation was observed between the mean net scores on the trials in the final two blocks (61–80 and 81–100) and the serum BDNF levels (Table 3). Table 4 shows the multiple regression analysis results for the mean net scores on each block of the trials. Depressive symptoms and the serum BDNF levels were significantly associated with the mean net scores on the trials in the final two blocks.

**Table 3:** **Correlation between the Iowa Gambling Task performance and serum BDNF, estimated IQ, and clinical symptoms**.

	**Mean amount of money earned (yen)**	**1~20**	**21~40**	**41~60**	**61~80**	**81~100**
Serum BDNF concentration (ng/ml)	0.06	0.02	0.03	0.09	0.24^*^	0.24^*^
CPZeq of total antipsychotic drugs(mg/day)	0.06	−0.05	−0.09	0.00	−0.1	−0.07
Estimate IQ	0.02	−0.06	−0.04	−0.01	−0.03	−0.06
PANSS-P	0.08	0.02	−0.08	0.00	0.02	0.02
PANSS-N	0.07	−0.03	−0.02	−0.1	−0.08	−0.02
PANSS-G	0.04	−0.03	−0.07	−0.01	−0.25^*^	−0.23^*^
PANSS-T	0.08	−0.01	−0.08	−0.04	−0.17	−0.13

*CPZ-eq, chlorpromazine-equivalent*.

* *p < 0.05*.

**Table 4:** **Multiple regression analysis results for the mean net scores on each block of the trials**.

	**Independent variables**	**Multiple regression statistic**			
		β	***SE***	***t*-value**	***p*-value**
IGT-1	Age	0.019	0.045	0.43	0.667
	CPZ-eq	−0.001	0.001	−0.62	0.535
	Depression	1.252	0.869	1.44	0.153
	PANSS-T	−0.459	0.051	−0.91	0.367
	Estimated-IQ	−0.399	0.060	−0.67	0.506
	Serum BDNF levels	0.009	0.070	0.13	0.894
IGT-2	Age	−0.050	0.054	−0.93	0.354
	CPZ-eq	−0.000	0.002	−0.21	0.831
	Depression	0.518	1.041	0.50	0.620
	PANSS-T	−0.049	0.06	−0.80	0.424
	Estimated-IQ	−0.039	0.071	−0.55	0.584
	Serum BDNF levels	0.019	0.084	0.23	0.819
IGT-3	Age	−0.117	0.056	−2.07	0.042^*^
	CPZ-eq	0.002	0.002	1.02	0.313
	Depression	0.834	1.084	0.77	0.444
	PANSS-T	−0.040	0.063	−0.63	0.532
	Estimated-IQ	−0.028	0.075	−0.37	0.710
	Serum BDNF levels	0.078	0.088	0.89	0.378
IGT-4	Age	−0.054	0.071	−0.75	0.453
	CPZ-eq	−0.000	0.002	−0.10	0.919
	Depression	−3.502	1.376	−2.54	0.013^*^
	PANSS-T	0.034	0.080	0.42	0.673
	Estimated-IQ	−0.017	0.095	−0.18	0.857
	Serum BDNF levels	0.23	0.112	2.06	0.043^*^
IGT-5	Age	−0.674	0.084	−0.81	0.423
	CPZ-eq	0.000	0.003	0.09	0.929
	Depression	−4.686	1.619	−2.89	0.005^*^
	PANSS-T	0.077	0.094	0.82	0.414
	Estimated-IQ	−0.458	0.111	−0.41	0.682
	Serum BDNF levels	0.269	0.131	2.05	0.044^*^

*PANSS-T, PANSS total score; CPZ-eq, chlorpromazine-equivalent; IGT-1: Card block 1-20; IGT-2: Card block 21-40; IGT-3: Card block 41-60; IGT-4: Card block 61-80; IGT-5: Card block 81-100*.

* *p < 0.05*.

## Discussion

A significant finding in the present study was that the serum BDNF levels and depressive symptoms correlated with decision-making. Several previous studies reported that schizophrenia patients exhibited diminished decision-making abilities compared to healthy individuals (Beninger et al., 2003; Ritter et al., 2004; Lee et al., 2007; Kim et al., 2009; Struglia et al., 2011). To the best of our knowledge, this is the first report to provide evidence suggesting that the serum BDNF levels reflect decision-making ability in patients with chronic schizophrenia. A recent meta-analysis revealed that the serum BDNF levels in schizophrenia patients are lower than those in healthy individuals (Green et al., 2011). In addition, recent studies have reported an association between poor performance on the IGT and the volume of several lesions in the hippocampus (Bonatti et al., 2009; Labudda et al., 2009; Yamano et al., 2011). The expression level of BDNF is normally high in the hippocampus. Taking these findings into account, diminished serum BDNF level in schizophrenia patients may reflect their poor decision-making performance. In short, serum BDNF may serve as a biomarker of decision-making ability in schizophrenia patients.

Wilder et al. reported that schizophrenia patients often selected the decks corresponding to infrequent and high-magnitude punishments (Wilder et al., 1998). Another study confirmed the finding that schizophrenia patients performed poorly compared to healthy individuals and often selected cards from the deck corresponding to high-magnitude punishments (Shurman et al., 2005). In the present study, the schizophrenia patients selected from good decks during the latter half of the task; however, optimizing the selection pattern appeared to be more difficult for the schizophrenia patients compared to the healthy individuals. Therefore, schizophrenia patients may tend to perform the same pattern of changes in the selection of the decks on the IGT as the control subjects. The magnitude of the occasional penalties has been reported to have little impact on the pattern of card selection.

The results in the present study are in accordance with those of previous publications (Beninger et al., 2003; Ritter et al., 2004; Shurman et al., 2005; Lee et al., 2007; Kim et al., 2009; Struglia et al., 2011). In addition, the present study revealed a significant correlation between decision-making performance and certain psychiatric symptoms. Previous studies have reported inconsistent results regarding the relationship between the IGT score and symptoms of schizophrenia, including a significant correlation to not only negative symptoms of schizophrenia (Shurman et al., 2005) but also positive symptoms of schizophrenia (Struglia et al., 2011). Several studies demonstrated an association between the serum BDNF levels and severity of major depressive disorder (Shimizu et al., 2003; Dell'Osso et al., 2010; Kurita et al., 2012; Yoshiumra et al., 2012). In contrast, no correlations existed between the serum BDNF levels and severity of depression (Karege et al., 2002b; Piccinni et al., 2008; Park et al., 2014). Taken together, it remains controversial that severity of a depressive state influences decision-making in schizophrenia patients. In short, it is not elucidated whether serum BDNF levels reflect or not depressive factors in schizophrenia. One study reported that depressive symptoms are associated with QOL in schizophrenia (Ueoka et al., 2011). Therefore, treatment targeting depressive symptoms may improve the QOL in schizophrenia patients.

The present study contained several limitations. First, the patients with schizophrenia were not classified into subtypes. Second, the schizophrenia patients were receiving various antipsychotic medications when the IGT was performed. Third, the number of subjects in the control group was small. Fourth, the schizophrenia patient group was receiving antipsychotics, a relevant problem because of the influence of the drugs on the BDNF levels. Fifth, since we evaluated the depressive symptoms in schizophrenia with only PANSS depressive items, we were poorly assessed and insufficient. Last, there is increasing evidence that sampling characteristics, several sociodemographic variables (such as age, sex, urbanicity, BMI), life-style factors (such as food, alcohol intake, and smoking status), somatic disease, and even self-reported depressive symptoms are relevant determinants of serum BDNF levels (Radka et al., 1996; Karege et al., 2002a; Bus et al., 2011). In conclusion, both depressive symptoms and the serum BDNF levels may be associated with the impairment of decision-making in schizophrenia patients.

## Author contributions

Dr. Hikaru Hori designed the study, performed the cognitive battery, collected the clinical data, performed the statistical analyses, wrote the first draft of the manuscript, and performed literature searches. Dr. Reiji Yoshimura and Dr. Jun Nakamura developed the study protocol and wrote the final manuscript. Dr. Asuka Katsuki performed the cognitive battery. Dr. Kiyokazu Atake collected the clinical data. All authors contributed to and approved the final manuscript.

### Conflict of interest statement

The authors declare that the research was conducted in the absence of any commercial or financial relationships that could be construed as a potential conflict of interest.
